# Metalloproteinase 1 downregulation in neurofibromatosis 1: Therapeutic potential of antimalarial hydroxychloroquine and chloroquine

**DOI:** 10.1038/s41419-021-03802-9

**Published:** 2021-05-19

**Authors:** Gaku Tsuji, Ayako Takai-Yumine, Takahiro Kato, Masutaka Furue

**Affiliations:** 1 Department of Dermatology, Graduate School of Medical Sciences, Kyushu University, Fukuoka, 812-8582 Japan; 2 Research and Clinical Center for Yusho and Dioxin, Kyushu University Hospital, Fukuoka, 812-8582 Japan; 3 Department of Neuropsychiatry, Graduate School of Medical Sciences, Kyushu University, Fukuoka, 812-8582 Japan; 4 Division of Skin Surface Sensing, Department of Dermatology, Graduate School of Medical Sciences, Kyushu University, Fukuoka, 812-8582 Japan

**Keywords:** Drug development, Mechanisms of disease

## Abstract

Neurofibromatosis type 1 is an autosomal dominant genetic disorder caused by mutation in the *neurofibromin 1* (*NF1*) gene. Its hallmarks are cutaneous findings including neurofibromas, benign peripheral nerve sheath tumors. We analyzed the collagen and matrix metalloproteinase 1 (MMP1) expression in Neurofibromatosis 1 cutaneous neurofibroma and found excessive expression of collagen and reduced expression of MMP1. To identify new therapeutic drugs for neurofibroma, we analyzed phosphorylation of components of the Ras pathway, which underlies NF1 regulation, and applied treatments to block this pathway (PD184352, U0126, and rapamycin) and lysosomal processes (chloroquine (CQ), hydroxychloroquine (HCQ), and bafilomycin A (BafA)) in cultured Neurofibromatosis 1 fibroblasts. We found that downregulation of the MMP1 protein was a key abnormal feature in the neurofibromatosis 1 fibroblasts and that the decreased MMP1 was restored by the lysosomal blockers CQ and HCQ, but not by the blockers of the Ras pathway. Moreover, the MMP1-upregulating activity of those lysosomal blockers was dependent on aryl hydrocarbon receptor (AHR) activation and ERK phosphorylation. Our findings suggest that lysosomal blockers are potential candidates for the treatment of Neurofibromatosis 1 neurofibroma.

## Introduction

Neurofibromatosis type 1 (Neurofibromatosis 1) is a genetic disorder that affects one in 2600 to 4500 live births^1,2^. Hallmarks of the disease are cutaneous findings including café au lait macules, skinfold freckling, and neurofibroma, a benign peripheral nerve sheath tumor^3–5^. Patients with neurofibromatosis 1 suffer from extensive extracutaneous lesions including optic glioma, Lisch nodule, scoliosis, bone involvement, and pseudoarthrosis. Neurofibromatosis 1 is also comorbid with neuronal complications such as learning difficulties, central nervous system tumors, and neurovascular diseases^3–6^. The clinical manifestations are variable, unpredictable, and potentially life-threatening. Malignant peripheral nerve sheath tumors are generally associated with a fatal outcome. They are also linked to disfigurement and social isolation, which cause deep psychological distress and reduce the quality of life of afflicted individuals^3–6^.

Cutaneous neurofibromas manifest as circumscribed tumors that are basically associated with nerves in the skin^7,8^. They can undergo a rapid initial proliferative phase, but then quickly become quiescent with extremely slow to no growth^3^. Multiple cutaneous and subcutaneous tumors adversely affect the quality of life^9^. Neurofibromatosis 1 results from an autosomal dominant loss of the *neurofibromin 1* (*NF1*) gene^10–12^. NF1 is a Ras GTPase activating protein and thus facilitates Ras inactivation^13,14^. In NF1-insufficient cells, Ras activation is not inhibited by NF1, which in turn upregulates prosurvival signaling via the PI3K-mTOR axis as well as transcriptional/proliferative signaling via the RAF-MEK-ERK pathway^3,14,15^.

Histopathological analysis has revealed that the cellular and extracellular composition of cutaneous neurofibroma is diverse, including Schwann-like cells, fibroblasts, perineural cells, and collagen matrix^7,16,17^. The collagen production is increased in cutaneous neurofibroma and the major collagen is type 1 collagen (COL1A1)^16,18,19^. Both PI3K-mTOR and RAF-MEK-ERK cascades regulate cell proliferation, DNA synthesis, apoptosis, and COL1A1 synthesis^3,14,15,20^. Recent year, sirolimus, a specific mTOR inhibitor has been used for the treatment of neurofibromatosis 1^21,22^. Although topical and systemic sirolimus is very efficacious for another mTOR-activating inherited genetic disorder, tuberous sclerosis^23,24^, sirolimus failed to reduce the neurofibroma volume in progressing and non-progressing neurofibromas^21,22^. The antifibrotic pirfenidone inhibits COL1A1 production in fibroblasts^25^ and is used for treating patients with idiopathic pulmonary fibrosis^26^. However, a clinical trial of pirfenidone failed to show its efficacy for neurofibromatosis 1^27^. These results indicate that a different approach may be necessary for this complex inherited disease.

Collagen deposition is regulated by the balancing of its production and degradation by matrix metalloproteinases (MMPs); MMP1 is the major enzyme degrading COL1A1^28^. However, few reports have demonstrated the expression of MMPs in neurofibromatosis 1. Walter et al. showed that increased stiffness of optic nerve tumor may be related to the downregulation of MMP2 in neurofibromatosis 1^29^. In addition, Muir reported that the expression of MMP1 and MMP-9 was increased in cultured cutaneous neurofibroma containing an abundance of Schwann cells^30^. However, to the best of our knowledge, no studies have focused on the expression of MMPs to treat cutaneous neurofibroma. In this study, we investigated the mRNA and protein expression of COL1A1 and MMP1 in dermal cell lines derived from neurofibromatosis 1 and healthy volunteers. We found that the downregulation of MMP1 protein was a key abnormal feature in the neurofibromatosis dermal cell lines and that this decrease in MMP1 was restored by the lysosomal blockers chloroquine (CQ) and hydroxychloroquine (HCQ)^31,32^. These antimalarial^33^ and antilupus drugs^34,35^ are thus potential candidates for the treatment of neurofibromatosis 1.

## Materials and methods

### Reagents and antibodies

Dermal fibroblast cells were cultured with Minimum Essential Eagle’s Medium (Sigma-Aldrich, St. Louis, MI, USA) supplied with nonessential amino acid solution (Thermo Fisher Scientific, Waltham, MA, USA), 15% fetal bovine serum (Nichirei Biosciences Inc., Tokyo, Japan), and antibiotics (Sigma-Aldrich). CQ and HCQ were purchased from Fujifilm (Osaka, Japan) and Tokyo Chemical Industry, Co., Ltd. (Tokyo, Japan), respectively, and dissolved in phosphate buffered saline. Bafilomycin A1 (Cayman Chemical, Ann Arbor, MI, USA), U0126 (Cell Signaling Technology, Danvers, MA, USA), PD184352 (Sigma-Aldrich), rapamycin (LC Laboratories, Woburn, MA, USA), AKT inhibitor (Abcam, Cambridge, UK), SB203580 (Selleck Chemicals, Houston, TX, USA), and SP600125 (Selleck Chemicals) were dissolved into dimethyl sulfoxide. Antibodies used in western blotting targeting β-actin (#13E5), MEK1/2, Akt, c-Raf, ERK, phospho-MEK1/2 (#41G9), phospho-Akt (#D9E), phospho-c-Raf (#56A6), phospho-ERK (#D13.14.4E), MMP1 (#E9S9N), and NF1 (#D7R7D), as well as anti-rabbit IgG HRP-linked antibody were purchased from Cell Signaling Technology. Antibody for Aryl hydrocarbon receptor (AHR, #H-211) was from Santa Cruz Biotechnology (Dallas, TX, USA) and that for collagen I (#EPR7785) was from Abcam. For immunohistochemical analysis, anti-collagen antibody (Nichirei Biosciences Inc.), anti-MMP1 antibody (Nichirei Biosciences Inc.), anti-S100 protein antibody (Nichirei Biosciences Inc.), AP-linked anti-rabbit antibody (Abcam), and rabbit isotype control antibody (BioLegend, San Diego, CA, USA) were used. Alexa Fluor488 goat anti-rabbit IgG H+L was purchased from Thermo Fisher Scientific.

### Study approval

This study was approved by the Ethics Committee of Kyushu University (#30-363 for immunohistological study and #24-132 for cell line establishment). Written informed consent was obtained from all of the volunteers. Punch biopsies were taken from a total of six donors, including three healthy controls and three patients diagnosed with neurofibromatosis type 1 (Supplemental Table 1) based on diagnostic criteria.

### Cell culture

To establish the human primary fibroblast cell culture, dermal fibroblast cells were isolated from biopsied skin tissue and cultured as described previously^36^. A lack of contamination of Schwann-like cells was confirmed by S100A protein staining. Before the experiments, cells were trypsinized and allowed to adhere to the culture plates for 24 h. Then, the cells underwent each experiment as detailed below. Each phosphorylation inhibitor was mixed with HCQ or vehicle in culture medium and then the cell culture medium was changed depending on the experimental conditions.

### NF1 genotyping

DNA was isolated from cultured fibroblast cells from a total of six donors with NucleoSpin Tissue (Qiagen, Hilden, Germany). DNA genotyping and data analysis were performed by Genewiz Inc. (South Plainfield, NJ, USA).

### Small interfering RNA transfection

Small interfering RNA (siRNA) targeting NF1 (s221793) or AHR (s1200) and scrambled RNA (Silence Negative Control No. 1) were purchased from Thermo Fisher Scientific. siRNA was transfected into fibroblast cells with lipofectamine RNAi Max (Thermo Fisher Scientific), following the manufacturer’s instructions.

### Quantitative real-time polymerase chain reaction (qRT-PCR)

Total RNA was isolated using RNase Mini Kit (Qiagen) and reverse-transcribed with Prime Script RT Reagent Kit (Takara Bio, Otsu, Japan), in accordance with the manufacturer’s instructions. The qPCR reactions were performed with the CFX Connect System (Bio-Rad Laboratories, Hercules, CA, USA) using TB Green Premix Ex Taq (Takara Bio). Cycling conditions comprised 95 °C for 30 s as the first step, followed by 40 cycles of qRT-PCR at 95 °C for 5 s and 60 °C for 20 s. mRNA expression was measured in triplicate wells and was normalized using β-actin as a housekeeping gene. The primer sequences are shown in Supplemental Table 2.

### Western blotting analysis

Cells were rinsed with ice-cold PBS and lysed with RIPA buffer containing protease inhibitor cocktail (Sigma-Aldrich) and PhosSTOP (Roche Diagnostics, Rotkreuz, Switzerland). Extracted proteins were denatured by boiling at 96 °C for 5 min with SDS-sample buffer containing 2-mercaptoethanol and loaded onto Blot 4–12% Bis-Tris Plus Gel (Thermo Fisher Scientific). The proteins transferred to a PVDF membrane (Merck Millipore, Burlington, MA, USA) were reacted with antibody diluted in Can Get Signal (Toyobo Co. Ltd., Osaka, Japan) and subjected to Super Signal West Pico (Thermo Fisher Scientific). Chemiluminescence was detected using ChemiDoc XRS (Bio-Rad) and densitometric analysis was performed.

### Enzyme-linked immunosorbent assay (ELISA)

The total cell culture supernatant was collected and frozen immediately at −80 °C until use. The concentration of secreted MMP1 was measured as manufacturer’s protocol (Boster Biological Technology, CA, USA; or R&D Systems).

### Immunohistofluorescence

Fibroblasts were seeded on µ-Slide 8-well chambers (ibidi, Gräfelfing, Germany) and harvested for 24 h. Treatment with 50 μM HCQ, 100 nM FICZ, or vehicle was applied for 6 h prior to immobilization with ice-cold acetone. Cells were blocked with 5% bovine serum albumin and reacted with primary antibodies at 4 °C overnight. Then, cells were reacted with Alexa Fluor488 secondary antibodies in the dark. Chambers were covered with cell-mounting medium containing DAPI (Santa Cruz) and images were taken with EVOS Cell Imaging Systems (Thermo Fisher Scientific).

### Immunohistochemistry

Skin biopsy samples were obtained from five patients and subjected to staining as per the protocol used at Kyushu University Hospital facilities as previously described^37^, with minor modification.

### Cell proliferation and viability test

Fibroblasts were seeded in a 96-well plate and harvested for 48 h. Viable cells were measured using a CCK-8 kit (Dojindo, Tokyo, Japan). The absorbance at 450 nm was measured with a microplate reader (Bio-Rad) and the analysis was performed in quadruplicate. To make a calibration curve for cell counting, a sequence of numbers of cells were seeded in a 96-well plate and allowed to adhere to the plate for 2 h. A standard curve was established following the manufacturer’s instructions. A calibration curve was made for each experiment. Cells at passages 3 to 4 were used to measure the cell proliferation rate.

### Statistical analysis

An unpaired two-tailed *t*-test and Tukey’s HSD test were applied as appropriate to evaluate statistical significance (*P < 0.05; **P < 0.01; ***P < 0.001). All analyses were performed using the JMP Pro software package (SAS Institute Japan Ltd., Tokyo, Japan).

## Results

### Downregulated expression of MMP1 in neurofibromas of neurofibromatosis 1 patients

**Fig. 1:**
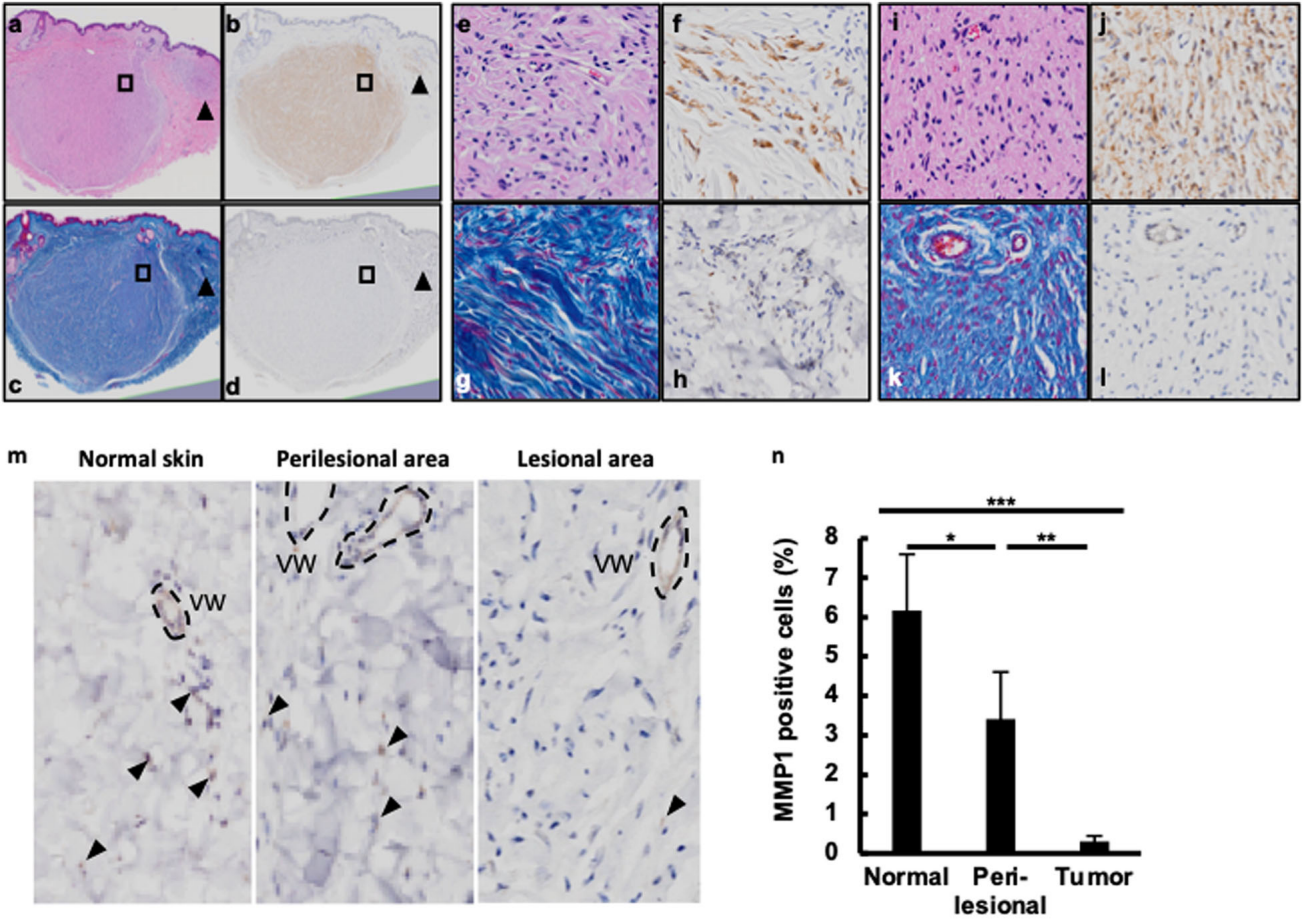
MMP1 expression was downregulated in cutaneous tumor in neurofibromatosis type 1 patients. Biopsy samples of cutaneous tumor were taken from the dermis of an NF1 patient and subjected to hematoxylin and eosin (**a**, **e**, **i**), S100 (**b**, **f**, **j**), Azan–Mallory (**c**, **g**, **k**), and MMP1 staining (**d**, **h**, **l**). Representative images of the tumor are shown in **a**–**d**, and magnified images of the squares in **a**–**d** are shown in **i**–**l**. The arrowheads show the early lesions of the peripheral regions, and their magnified images are shown in **e**–**h**. **m**, **n** Cutaneous biopsy samples of normal skin, perilesional normal skin, and tumor lesion were taken from five NF1 patients, on which MMP1 staining was performed. Arrowhead: MMP1-expressing cells. VW: blood vessel wall. **n** MMP1-positive cells were counted from three images of each section (normal skin, perilesional normal skin, and tumor); mean (with SEM) percentage of positive cells from five patients is shown (*n* = 5).

To evaluate MMP1 expression in neurofibromas, we first conducted immunohistochemical analysis in neurofibromas of five neurofibromatosis 1 patients. As neurofibroma is characterized by large numbers of S100 + Schwann-like cells and fibroblasts^7,16^, we utilized a specific antibody for S100 protein to clarify the lesional skin of neurofibroma. Azan–Mallory staining, which stains collagenous fibers deep blue and glial cells and neurons reddish purple, was also utilized. The lesional skin of the neurofibromas contained S100 + cells (Fig. 1b, j, stained brown) with neo-collagen accumulation, which were weakly stained blue with Azan–Mallory staining (Fig. 1c, k). The majority of stromal cells in the neurofibroma lesions were virtually negative for MMP1 staining (Fig. 1d, i). Even in the early-stage lesions of neurofibroma (Fig. 1e–h), the stromal cells were MMP1-negative (Fig. 1h). We then counted the stromal MMP1-positive cells in the neurofibromas (lesional and perilesional areas) and five samples of normal healthy skin. Dermal vascular endothelial cells were MMP1-positive and served as a positive control (Fig. 1m). The proportions of MMP1-positive stromal cells were 6.2 ± 1.4% (mean ± standard error), 3.4 ± 1.3%, and 0.3 ± 0.1% in normal control skin, perilesional area of neurofibromas, and lesional area of neurofibromas, respectively (Fig. 1n).

### Downregulated expression of MMP1 in cultured fibroblast cells from neurofibromatosis 1 patients

**Fig. 2:**
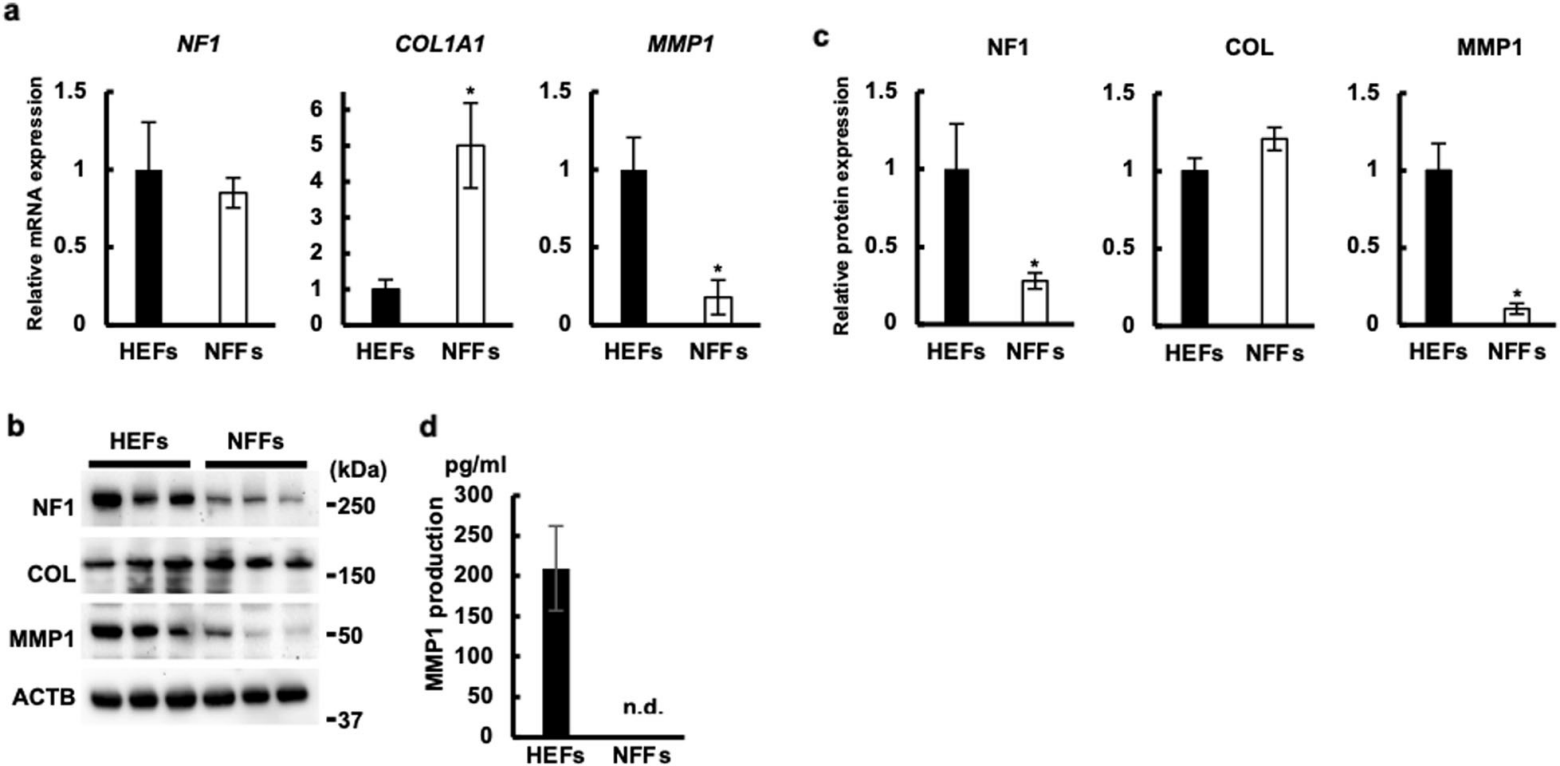
MMP1 protein expression was downregulated in cultured fibroblasts derived from NF1 patients. Three cell lines of each of HEFs and NFFs were cultured in triplicate wells for 48 h. **a** Relative mRNA expression of the indicated genes is shown. Black bars represent expression in HEFs and white bars represent expression in NFFs. **b**, **c** Expression of the indicated proteins was analyzed by western blot. Representative images of western blot are shown in **b** and relative expression normalized to β-actin is shown in **c**. **d** MMP1 production for 24 h was measured. All cell lines were cultured in triplicate wells and the mean ± SEM of HEFs or NFFs is shown.

To further characterize the biological response of neurofibroma cells, we established three primary dermal fibroblastic cell lines from neurofibromatosis 1 patients (NFFs) and three normal primary fibroblastic cell lines from healthy control skin (HEFs), as reported previously^36^ (Supplementary Table 1). All three NFFs revealed stop codon mutations in the *NF1* gene heterozygously (Supplementary Table 1). Other exonic mutations in NFFs (2034 G > A, 702 G > A) were also detected in the healthy donors. There was no difference in morphology or proliferative capacity between HEFs and NFFs (Supplementary Fig. S1). Although the mRNA expression of *NF1* was comparable between HEFs and NFFs (Fig. 2a), the protein expression of NF1 was significantly downregulated in NFFs compared with that in HEFs (Fig. 2b, c). The protein expression of COL1A1 was comparable between HEFs and NFFs, while the expression of MMP1 protein was significantly decreased in NFFs compared with that in HEFs, as revealed by western blot analysis (Fig. 2b, c). In parallel with this, significant amounts of MMP1 protein were detected in the supernatants of HEFs, while NFFs did not release detectable amounts of it (Fig. 2d). Although the RAF-MEK-ERK and PI3K-AKT-mTOR cascades have been reported to be accelerated in neurofibromatosis 1^3,14,15^, we could not detect significant differences in the phosphorylation levels of RAF, MEK, ERK, and AKT between HEFs and NFFs (Supplementary Fig. S2). These findings suggested that the cultured NFFs recapitulated the biological nature of stromal cells of neurofibromas, at least in terms of the NF1 and MMP1 downregulation.

### Downregulated expression of MMP1 is restored by CQ and HCQ

To investigate the effects of the RAF-MEK-ERK and PI3K-AKT-mTOR axes on the MMP1 downregulation in NFFs, we treated NFFs with the ERK cascade inhibitors U0126 or PD184352 or rapamycin, mTOR inhibitor. Their inhibitory effects were confirmed by the findings that U0126 and PD184352 inhibited the phosphorylation of ERK (Supplementary Fig. S3b, c), while rapamycin led to the accumulation of phosphorylated AKT (Supplementary Fig. S3f, g). However, U0126, PD184352, and rapamycin could not restore the MMP1 downregulation, but instead exacerbated it (Supplementary Fig. S3b, d, g, h). COL1A1 protein expression was not altered by U0126 or PD184352 (Supplementary Fig. S3b, e), and was decreased by rapamycin (Supplementary Fig. S3f, i). These results indicated that the reported conventional pathogenic pathways were not involved in the MMP1 downregulation in NFFs.

**Fig. 3:**
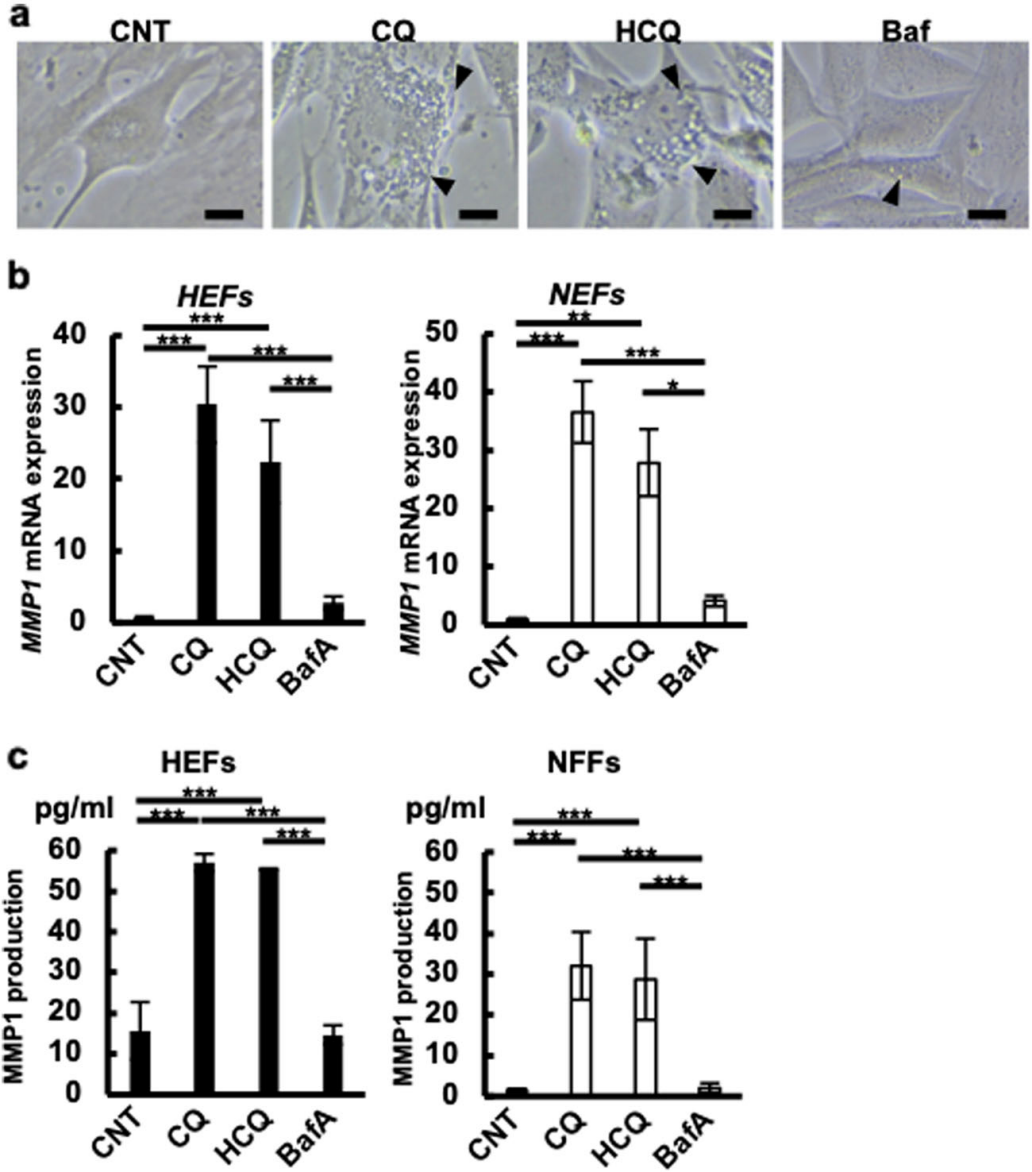
Lysosomotropic agents upregulated MMP1 expression. Three cell lines of each of HEFs and NFFs were cultured in triplicate wells and treated with 50 μM CQ, 50 μM HCQ, 5 nM BafA, or vehicle. **a** Representative cell morphology of NFFs treated with the indicated drugs or vehicle. Arrowheads indicate vesicles. Scale bar indicates 1 μm. **b** Relative MMP1 mRNA expression in 24 h of treatment is shown. **c** MMP1 proteins in culture medium upon 48 h of treatment with lysosomotropic agents were analyzed by ELISA. Black bar indicates expression in HEFs and white bar indicates expression in NFFs (**b**, **c**). The data represented the mean ± SEM of the three independent experiments.

As MMP1 is degraded in lysosomes^38^, we next examined the effects of the lysosomal inhibitors CQ, HCQ, and bafilomycin A (BafA)^31,32,34^ on the MMP1 expression in HEFs and NFFs. The effects of CQ and HCQ were confirmed by them inducing numerous intracellular vesicles due to lysosomal swelling, compared with the findings in the vehicle control (Fig. 3a). Lysosomal swelling was only weakly observed in BafA-treated cells (Fig. 3a). Notably, CQ and HCQ significantly increased the mRNA (Fig. 3b) and protein (Fig. 3c and Supplementary Fig. S4) levels of MMP1 compared with the findings for the vehicle control in both HEFs and NFFs. The MMP1 proteins upregulated by CQ and HCQ were actually released in the culture supernatants (Fig. 3c). HCQ is more applicable for clinical use than CQ because the major associated adverse event, retinopathy, occurs less with HCQ than with CQ^34^. Therefore, we mainly used HCQ rather than CQ in the following experiments.

### CQ- and HCQ-mediated MMP1 upregulation is not related to NF1 protein level

**Fig. 4:**
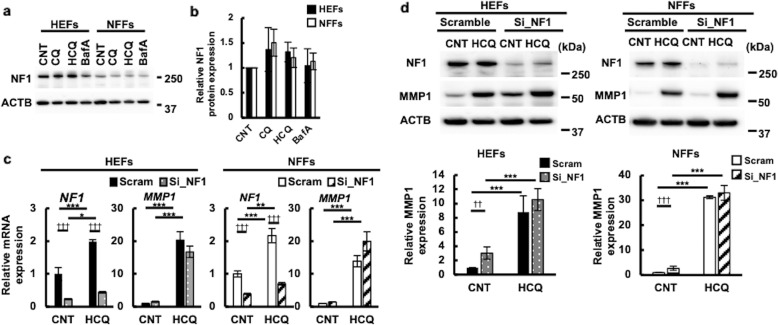
MMP1-inducing activity of HCQ was not dependent on neurofibromin protein. **a**, **b** Three cell lines of each HEFs and NFFs were treated with 50 μM CQ, 50 μM HCQ, or 5 nM BafA for 48 h and NF1 expression was analyzed by western blotting. Representative images from three independent experiments are shown in **a**. NF1 expression was normalized with β-actin and relative expression levels were calculated considering the intensity of vehicle-treated sample as 1 and shown in **b**. The data represented the mean ± SEM of the three independent experiments. **c**, **d** The HEFs (KYU168) and NFFs (KYU101) were transfected with scrambled RNA or si_NF1, and treated with 50 μM HCQ. **c** NF1 and MMP1 mRNA expression in 24 h is shown. The data represented the mean ± SD of the three independent experiments. **d** NF1, MMP1, and β-actin expression was analyzed by western blot and representative images from three independent experiments are shown in the upper part. Relative MMP1 expression is shown in the lower part. The data represented the mean ± SD of the three independent experiments. *: compared among drug-treated groups. †: compared with scrambled RNA-transfected group.

As NFFs had reduced levels of NF1, we assumed that CQ and HCQ might increase these levels, subsequently restoring the MMP1 downregulation. Indeed, the expression levels of NF1 protein tended to be upregulated by CQ and HCQ in HEFs and NFFs, but this did not reach statistical significance (Fig. 4a, b). For further analysis, we used KYU168 and KYU101 as representatives of HEFs and NFFs, respectively, since KYU168 and KYU101 did not differ from other HEFs and NFFs in terms of the findings of morphological observation, proliferation analysis, and expression analysis of NF1 and MMP1 mRNA and proteins, while KYQ403 and KYQ404 did not proliferate upon more than six subcultures. To determine the relationship between NF1 and MMP1 levels, we measured the mRNA and protein levels of MMP1 in HEFs and NFFs transfected with NF1 siRNA or control scrambled siRNA. The transfection of NF1 siRNA successfully lowered the mRNA (Fig. 4c) and protein (Fig. 4d) levels of NF1 in both HEFs and NFFs. Unexpectedly, NF1 knockdown rather augmented the MMP1 expression in both HEFs and NFFs (Fig. 4d). Considering the reduced MMP1 expression in NF1-insufficient NFFs, NF1 deficiency did not directly cause the MMP1 downregulation. In accordance with this, HCQ-mediated upregulation of MMP1 was not affected by the transfection of NF1 siRNA (Fig. 4c, d). These results highlight the possibility that HCQ actively upregulates MMP1 irrespective of the cellular NF1 level.

### HCQ-mediated MMP1 upregulation is dependent on AHR

**Fig. 5:**
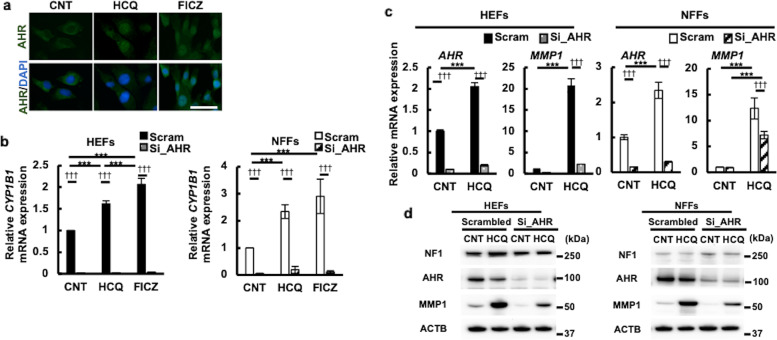
HCQ-induced MMP1 through AHR activation. **a** HEFs were treated with 50 μM HCQ for 6 h or 100 nM FICZ for 1 h and stained with anti-AHR antibody. Nuclei were stained with 4,6-diamidino-2-phenylindole (DAPI). Scale bar, 50 μm. **b**–**d** HEFs (KYU168) and NFFs (KYU101) were transfected with scrambled RNA or si_AHR and treated with 50 μM HCQ. **b** *CYP1B1* mRNA expression in 24 h is shown. **c** *AHR* and *MMP1* mRNA expression in 24 h is shown. The data represented the mean ± SD of the three independent experiments (**b**, **c**). **d** NF1, AHR, MMP1, and β-actin expression was analyzed by western blot and representative images from three independent experiments are shown. * compared among drug-treated groups. † compared with scrambled RNA-transfected group.

AHR ligands such as 6-formylindolo[3,2b]carbazole (FICZ) are known to upregulate the mRNA and protein levels of MMP1^39^. When activated, cytoplasmic AHR is translocated into the nucleus^40,41^ and upregulates the transcription of target genes such as *CYP1B1* in fibroblasts^37,39^. We next examined whether HCQ serves as an AHR ligand. Compared with the predominantly cytoplasmic staining of AHR in the untreated control NFFs, HCQ appeared to induce the cytoplasmic-to-nuclear translocation of AHR (Fig. 5a). In addition, HCQ significantly augmented the *CYP1B1* gene expression in both HEFs and NFFs, as did FICZ (positive control) (Fig. 5b). The HCQ-induced *CYP1B1* upregulation was canceled in HEFs and NFFs transfected with AHR siRNA (see Fig. 5c, d, for knockdown efficiency), indicating that HCQ is an AHR ligand.

We next investigated the effects of AHR knockdown on the HCQ-induced MMP1 upregulation. The mRNA and protein expression of AHR was successfully decreased by transfection with AHR siRNA compared with that with control scrambled siRNA in both HEFs and NFFs (Fig. 5c, d). Notably, the HCQ-induced MMP1 upregulation was reduced in both HEFs and NFFs transfected with AHR siRNA (Fig. 5c, d). In addition, baseline MMP1 expression was also decreased by AHR siRNA transfection (Fig. 5c). The NF1 protein levels were not significantly affected by AHR knockdown (Fig. 5d).

### HCQ-induced MMP1 upregulation is mediated by ERK pathway

**Fig. 6:**
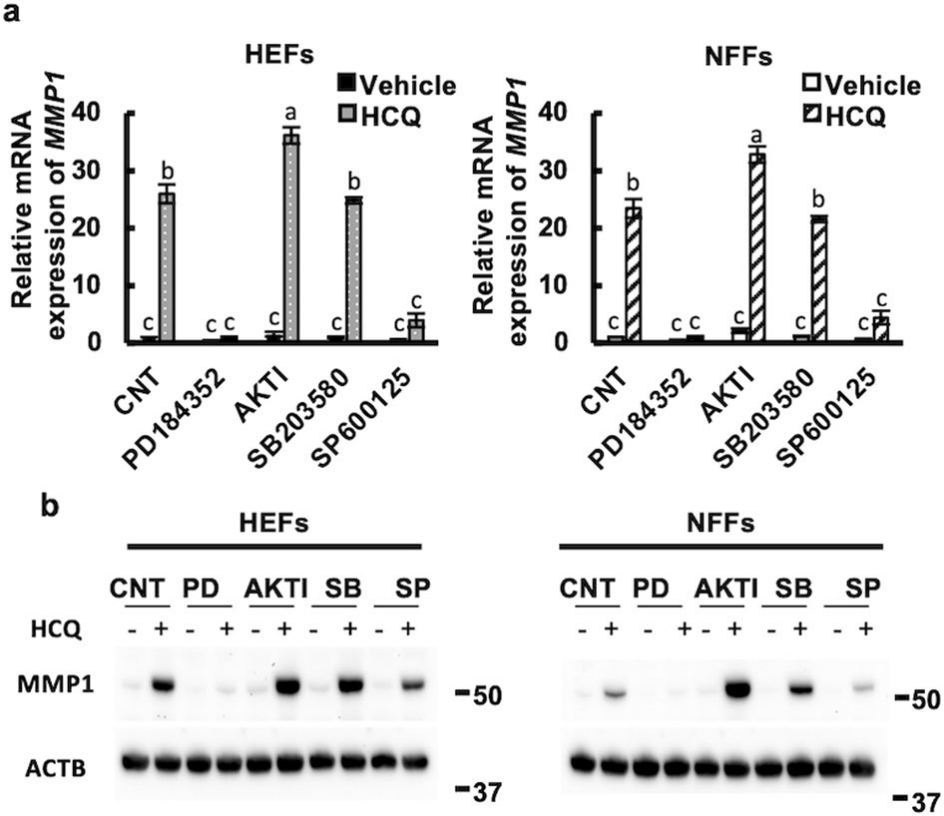
ERK phosphorylation is crucial for MMP1-inducing activity of HCQ. HEFs (KYU168) and NFFs (KYU101) were treated with 50 μM HCQ and phosphorylation inhibitors of the MAPK pathway for 24 h; MMP1 mRNA (**a**) and protein (**b**) expression is shown. PD: 10 μM PD184352 (ERK inhibitor), AKTI: 5 μM (AKT inhibitor), SB: 10 μM SB203580 (p38 MAPK inhibitor), and SP: 10 μM SP600125 (JNK inhibitor). The data represented the mean ± SD of the three independent experiments (**a**). The different letters (a, b, c) above the bars indicate statistically significant differences (*P* < 0.05).

The involvement of AHR activation and ERK pathway in fibroblasts^42^, dendritic cells^43^, adipocytes^44^, and keratinocytes^45^ has been reported, including by our group. To further investigate the signaling pathway governing HCQ-induced MMP1 upregulation, we treated the HEFs and NFFs with HCQ in the presence and absence of an ERK inhibitor (PD184352), AKT inhibitor (AKTI), p38 MAPK inhibitor (SB203580), or JNK inhibitor (SP600125). The upregulation of HCQ-induced MMP1 mRNA (Fig. 6a) and protein (Fig. 6b) was completely inhibited by the ERK inhibitor and partially by the JNK inhibitor in HEFs and NFFs. However, it was not inhibited by either the AKT inhibitor or the p38 MAPK inhibitor (Fig. 6a, b). Considering the robust inhibition of MMP1 expression by the ERK inhibitor, HCQ mainly signals through the AHR-ERK axis and upregulates MMP1 expression in both HEFs and NFFs. These results coincide with a previous report describing that FICZ-induced MMP1 upregulation is mediated by the AHR-ERK pathway^41^.

## Discussion

The number of cutaneous neurofibromas generally increases with age in patients with neurofibromatosis 1^46,47^. According to an epidemiological study by Ehara et al., the average number of cutaneous neurofibromas reaches 155 in the 30 s, 1053 in the 40 s, and 2040 in the 70 s in patients with neurofibromatosis 1. A gradual increase in the number of cutaneous neurofibromas occurs in 61% of patients^46^. The disfigurement caused by cutaneous neurofibromas significantly deteriorates the quality of life of afflicted individuals^3–6^.

Loss-of-function mutation of the *NF1* gene is the major genetic cause of neurofibromatosis 1^10–12^. Functional insufficiency of NF1 protein is known to lead to excessive activation of the PI3K-mTOR and RAF-MEK-ERK signaling pathways^3,13–15^. Although these pathways are believed to induce the proliferation of neurofibroma cells and COL1A1 production, leading to neurofibroma formation, the therapeutic outcomes of the specific mTOR inhibitor sirolimus and the anti-collagenogenic pirfenidone in clinical trials have not been satisfactory^21,22^.

COL1A1 deposition is an integral part of neurofibroma formation^16,18^. As the anti-collagenogenic pirfenidone is not effective for treating cutaneous neurofibromas in neurofibromatosis 1^27^, we hypothesized that the collagenolytic process, instead of the collagenogenic process, may be disturbed in this disease. As MMP1 is the major enzyme degrading COL1A1^39,41,48^, we first focused on its immunohistological expression in cutaneous neurofibromas. Notably, the number of MMP1 + stromal cells was significantly reduced in the lesional area of cutaneous neurofibromas compared with that in the perilesional area of neuromas or in normal control skin (Fig. 1n). In vitro experiments revealed that the three NFFs obtained from three independent neurofibromatosis 1 patients exhibited point mutations of the NF1 gene, which result in reduced NF1 protein expression compared with that in HEFs established from healthy volunteers. Two NFF cell lines, KYU403 and KYU404, have the same point mutation of 5905 C > T (Supplemental Table 1) showing limited proliferative ability, suggesting that 5905 C > T is a crucial mutation affecting long-term cell mortality. Similar to neurofibromas in vivo, MMP1 levels were significantly reduced in NFFs compared with those in HEFs in vitro (Fig. 2c, d). The COL1A1 production in NFFs was comparable with that in HEFs (Fig. 2c, d).

Agents with the potential to restore MMP1 downregulation may have therapeutic value in neurofibromatosis 1. However, specific inhibitors against the PI3K-mTOR (rapamycin) and RAF-MEK-ERK (U0126 and PD184352) axes failed to restore the reduced MMP1 expression in NFFs (Supplemental Fig. S3). This result might explain why the effect of oral rapamycin treatment on neurofibromatosis was modest in a clinical study^22^. In addition, this result is partially consistent with a previous report describing that MEK-ERK activation is involved in the induction of MMP1 expression in human breast adenocarcinoma cell lines^49^.

As the degradation of MMP1 occurs in the lysosomes, we next examined the effects of CQ and HCQ, which are lysosomal inhibitors on NFFs^31,32,34^. These antimalarial drugs accumulate in the lysosomes and inhibit the endocytotic, phagocytotic, and autophagocytotic processes by increasing the pH, which prevents the activity of lysosomal enzymes^34^. Notably, CQ and HCQ raised the mRNA and protein levels of MMP1 and accelerated its release in the culture supernatants in HEFs and NFFs (Fig. 3 and Supplemental Fig. S4). As the NF1 knockdown by NF1 siRNA did not influence the baseline and HCQ-mediated upregulation of MMP1 expression in both HEFs and NFFs, this MMP1-upregulating effect by HCQ operates irrespective of the intracellular NF1 level.

Since induction of the mRNA and protein expression of MMP1 is highly regulated by AHR-ERK signaling^39,41^, we next investigated the possibility that HCQ may activate AHR. The results showed that this was indeed the case. HCQ induced the nuclear translocation of AHR and induced transcription of the AHR target gene *CYP1B1*. In addition, AHR knockdown reduced the HCQ-mediated MMP1 upregulation.

**Fig. 7:**
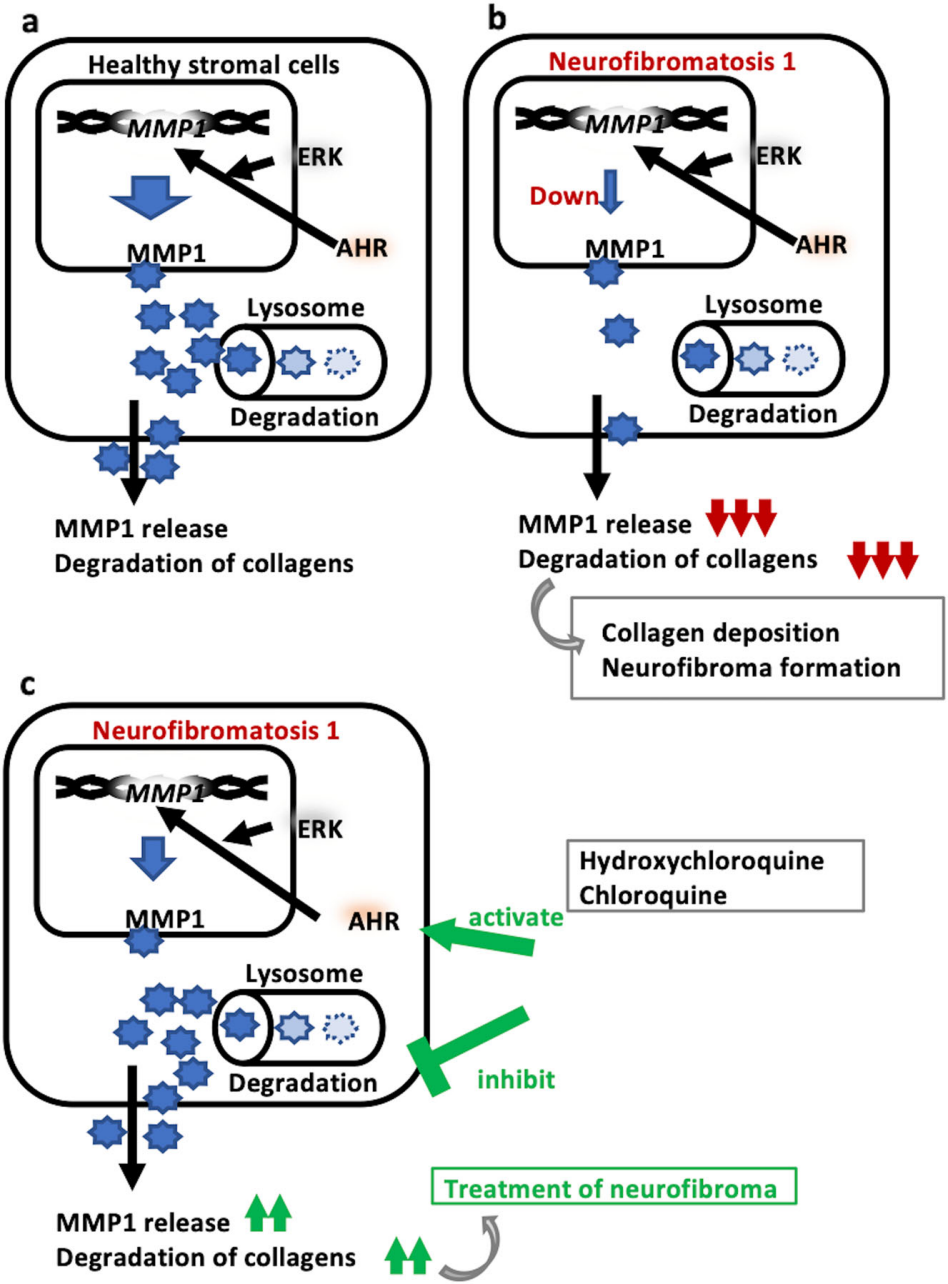
Schematic image of MMP1 induction by HCQ in neurofibromatosis type 1 patients. Schematic summarizing of MMP1 induction by HCQ and CQ in NFFs. **a**, **b** MMP1 reduction reads collagen accumulation and generates or worsen neurofibromas in neurofibromatosis 1 patients. **c** AHR activated by HCQ or CQ induces ERK phosphorylation and MMP1 expression. At the same time, HCQ and CQ inhibit lysosomal degradation of MMP1.

Based on these findings, we propose the following hypothesis. In healthy stromal cells (Fig. 7a), the mRNA and protein expression of MMP1 is mainly dependent on the AHR-ERK pathway. Some MMP1 proteins are degraded in the lysosomes, while some of them are released into the stroma and degrade collagens. In neurofibromatosis 1 (Fig. 7a), the mRNA and protein expression of MMP1 is markedly downregulated by an unknown mechanism(s). Its downregulation is not directly linked to a decreased level of NF1 proteins. The antimalarial drugs HCQ and CQ activate the AHR-ERK pathway and enhance the mRNA and protein expression of MMP1 (Fig. 7c). In addition, HCQ and CQ inhibit the lysosomal degradation process, which further increases the level of MMP1 protein. An excess of MMP1 protein is released even in neurofibromatosis 1 cells, and may restore the collagen-degrading capacity and be useful for the treatment of neurofibromas.

To date there are no effective therapeutic drugs available to modulate the progression of neurofibroma for adult neurofibromatosis 1 patients. As we show here, CQ and HCQ can be promising drugs for neurofibroma to decrease the collagen component, which is overexpressed in cutaneous and non-cutaneous lesions, through an increase in MMP1. However, there is concern about adverse effects associated with the treatment of CQ and HCQ^50,51^. Retinopathy and corneal deposits are widely known adverse effects of these drugs. Although corneal deposits can be reversed by discontinuing these drugs, retinopathy can cause irreversible vision loss^51^. However, retinopathy can be prevented by observing the maximum daily dosage based on ideal body weight. Additionally, ongoing monitoring is important to find this symptom at the earliest point of potential damage. Considering that neurofibromatosis 1 patients suffer from cutaneous and non-cutaneous neurofibroma, which decreases the quality of life, it is worth pursuing the use of CQ and HCQ to improve the outcome of those patients.

In conclusion, the mRNA and protein expression of MMP1 is markedly reduced in stromal cells in neurofibromatosis 1. This feature, though not directly related to the decrease in NF1 proteins, may be involved in the collagen accumulation in neurofibroma. The antimalarial drugs HCQ, CQ are feasible options to restore the MMP1 production and release in stromal cells in neurofibromatosis 1 by dual mechanisms, one by activating the AHR-ERK-MMP1 pathway and the other by inhibiting the lysosomal degradation of MMP1 proteins. The AHR-activating antimalarial drugs are potentially applicable for treating the devastating cutaneous neurofibromas in neurofibromatosis 1.

## Supplementary information

Supplementary Table 1

Supplementary Table 2

Supplementary text

Supplemental Figure S1

Supplemental Figure S2

Supplemental Figure S3

Supplemental Figure S4
