# Supplementary material for: Metalloproteinase 1 downregulation in neurofibromatosis 1: Therapeutic potential of antimalarial hydroxychloroquine and chloroquine
Source: Cell Death Dis. 2021 May 19;12(6):513. doi: 10.1038/s41419-021-03802-9 (PMC8134427; doi:10.1038/s41419-021-03802-9)
Supplement: Supplementary file 1 — Supplementary Table 1 [file 41419_2021_3802_MOESM1_ESM.docx]

**Supplemental Table 1.** Characteristics of fibroblast cell lines used in this study.

|  | ID | Type | Sex | Age | Exonic *NF1* gene mutation | | Effect of mutation |
| --- | --- | --- | --- | --- | --- | --- | --- |
| HEFs Dermal cell lines from healthy volunteers | KYU106 | Healthy | M | 69 | 2034G>A | Homozygous | Synonymous |
|  | KYU168 | Healthy | M | 61 | 702G>A | Heterozygous | Synonymous |
|  |  |  |  |  | 2034G>A | Heterozygous | Synonymous |
|  |  |  |  |  | 5160G>T | Heterozygous | E1720D |
|  | KYU176 | Healthy | M | 49 | 702G>A | Heterozygous | Synonymous |
|  |  |  |  |  | 2034G>A | Heterozygous | Synonymous |
| NFFs Dermal cell lines from neurofibromatosis 1 | KYU101 | NF1 | F | 67 | 2034G>A | Homozygous | Synonymous |
|  |  |  |  |  | 3143G>A | Heterozygous | Stop gain |
|  | KYQ403 | NF1 | M | 52 | 702G>A | Homozygous | Synonymous |
|  |  |  |  |  | 5905C>T | Heterozygous | Stop gain |
|  | KYQ404 | NF1 | M | 57 | 702G>A | Homozygous | Synonymous |
|  |  |  |  |  | 5905C>T | Heterozygous | Stop gain |
