# Supplementary material for: Metalloproteinase 1 downregulation in neurofibromatosis 1: Therapeutic potential of antimalarial hydroxychloroquine and chloroquine
Source: Cell Death Dis. 2021 May 19;12(6):513. doi: 10.1038/s41419-021-03802-9 (PMC8134427; doi:10.1038/s41419-021-03802-9)
Supplement: Supplementary file 2 — Supplementary Table 2 [file 41419_2021_3802_MOESM2_ESM.docx]

**Supplementary Table 2.** Primer pairs used in this study.

| Gene | Forward primer (5’-3’) | Reverse primer (5’-3’) |
| --- | --- | --- |
| *ACTB* | attgccgacaggatgcaga | gagtacttgcgctcaggagga |
| *COL1A1* | gcttggtccacttgcttgaaga | gagcattgcctttgattgctg |
| *MMP1* | aaggtggaccaacaatttcaga | tgaaggtgtagctagggtacatcaa |
| *NF1* | agatgaaacgatgctggtcaaa | cctgtaacctggtagaaatgcga |
| *AHR* | caaatccttccaagcggcata | cgctgagcctaagaactgaaag |
| *CYP1B1* | tggctgctcctcctcttc | ggctggtcacccatacaa |
