# Supplementary material for: Metalloproteinase 1 downregulation in neurofibromatosis 1: Therapeutic potential of antimalarial hydroxychloroquine and chloroquine
Source: Cell Death Dis. 2021 May 19;12(6):513. doi: 10.1038/s41419-021-03802-9 (PMC8134427; doi:10.1038/s41419-021-03802-9)
Supplement: Supplementary file 3 — Supplementary text [file 41419_2021_3802_MOESM3_ESM.docx]

**Supplemental Figure S1. Characteristics and proliferative capability of established HEFs and NFFs. a**, **b** Typical morphology of HEFs (**a**) and NFFs (**b**). (**c**) Three cell lines of each of NEFs and NFFs were cultured in triplicated, the proliferative capacity was measured in 24 h, and the proliferation rate was calculated as fold change. Data represent mean ± SEM of HEFs or NFFs of three independent experiments.

**Supplemental Figure S2. Phosphorylation of ERK was upregulated in NFFs.** Three cell lines of each of HEFs and NFFs were cultured in triplicate wells and phosphorylation of ERK and AKT pathway proteins was analyzed by western blot. **a** Representative images of HEFs and NFFs are shown. **b** Relative phosphorylation levels are presented as mean ± SEM of the three independent experiments.

**Supplemental Figure S3 Inhibition of Ras pathway decreased the MMP1 protein expression in NFFs**. Three different cell lines of NFFs were cultured in triplicate wells and subjected to each experiment. **a** Schematic image of NF1 and inhibition of downstream pathway of Ras. **b-i** NFFs were treated with 10 μM U0126, 10 μM PD184352 (PD), 10 μM rapamycin (RAP) or vehicle (CNT) for 24 h. Phosphorylation of ERK and AKT and expression of COL1A1 and MMP1 proteins in whole-cell lysate were analyzed by western blotting. **b,f** Representative images from three independent experiments are shown. Each protein expression was normalized with β-actin and relative phosphorylation level (**c**, **g**) and protein expression (**d**, **e**, **h**, **i**) were calculated considering the intensity of vehicle-treated samples as 1. Relative phosphorylation and protein expression levels are presented as mean ± SEM of the three independent experiments.

**Supplemental Figure S4. MMP1 protein induction by lysosomotropic agents.** The HEFs (KYU168) and NFFs (KYU101) were treated with 50 μM CQ, 50 μM HCQ, 5 nM BafA, or vehicle (CNT). Representative images from three independent experiments are shown in the upper panel and normalized MMP1 expression is shown in the lower graphs. The data represented the mean ± SD of the three independent experiments.
